# Assessing the toxicity of Pb- and Sn-based perovskite solar cells in model organism *Danio rerio*

**DOI:** 10.1038/srep18721

**Published:** 2016-01-13

**Authors:** Aslihan Babayigit, Dinh Duy Thanh, Anitha Ethirajan, Jean Manca, Marc Muller, Hans-Gerd Boyen, Bert Conings

**Affiliations:** 1 Hasselt University, Institute for Materials Research, Wetenschapspark 1, 3590 Diepenbeek, Belgium; 2 University of Liège, Laboratory for Organogenesis and Regeneration, GIGA-Research, B34, Avenue de l’Hôpital 1, 4000 Sart-Tilman, Belgium; 3 Hasselt University, X-LaB, Agoralaan 1, Building D, 3590 Diepenbeek, Belgium

## Abstract

Intensive development of organometal halide perovskite solar cells has lead to a dramatic surge in power conversion efficiency up to 20%. Unfortunately, the most efficient perovskite solar cells all contain lead (Pb), which is an unsettling flaw that leads to severe environmental concerns and is therefore a stumbling block envisioning their large-scale application. Aiming for the retention of favorable electro-optical properties, tin (Sn) has been considered the most likely substitute. Preliminary studies have however shown that Sn-based perovskites are highly unstable and, moreover, Sn is also enlisted as a harmful chemical, with similar concerns regarding environment and health. To bring more clarity into the appropriateness of both metals in perovskite solar cells, we provide a case study with systematic comparison regarding the environmental impact of Pb- and Sn-based perovskites, using zebrafish (*Danio Rerio*) as model organism. Uncovering an unexpected route of intoxication in the form of acidification, it is shown that Sn based perovskite may not be the ideal Pb surrogate.

Within the branch of photovoltaic solar energy, crystalline silicon solar cells have been the prevailing technology for more than half a century. While the production process of this type of solar cells has been thoroughly optimized, there is a fundamental bottom limit to their energy payback time, as their fabrication requires high temperatures^1^,^2^. Unfortunately, even despite significant research efforts in organic solar cells, dye-sensitized solar cells (DSSC) and hybrid solar cells, these technologies are not yet sufficiently mature to qualify as potential competitors for silicon technology, mainly due to relatively low efficiencies and limited durability^3^,^4^,^5^,^6^. A paradigm shift was heralded in 2009, when organometal halide perovskite crystals were first reported as light absorbers in solar cells by Miyasaka *et al.*, a material class that was later discovered to possess virtually all of the desirable properties to compete with silicon^7^. Perovskite solar cells trumping organic and hybrid solar technology so quickly in terms of power conversion efficiency proclaimed a new era for third generation photovoltaics and spurred many researchers to study and tune these solar cells, resulting in remarkable advances on a short timescale (>20% power conversion efficiency in 2015) and making perovskite solar cells the fastest developing photovoltaic technology of all time^8^,^9^,^10^,^11^,^12^. There are, however, a few stumble stones that currently prevent perovskite technology to become ready for large-scale applications. The first one is material instability. The organometal halide perovskites successfully employed in solar cells so far are prone to degrade due to stress factors like prolonged UV irradiation, oxygen and slightly elevated temperatures, all as a consequence of their low formation energy^13^,^14^,^15^,^16^. The second obstacle is that the most suitable metal cation for highly efficient perovskite solar cells is lead (Pb^2+^), which gives rise to apprehension from an environmental standpoint^17^,^18^,^19^,^20^,^21^,^22^. Even though strategies are being developed to decelerate or circumvent degradation, another caveat is that the strong hydrophilicity of the currently typically used organic cations makes perovskite sensitive to humidity^16^,^23^,^24^. Structural failure of an outdoor perovskite module, exposing the perovskite to the elements, would therefore still result in the disintegration of its unit cells and commensurate release of Pb-containing compounds into the environment. Surely, there are also concerns about the potential environmental burden upon both occupational and non-occupational exposure during fabrication and disposal^18^,^20^. Many proposed the replacement of Pb by tin (Sn) in order to render perovskite light absorbers less toxic or even nontoxic^19^,^25^,^26^,^27^,^28^. Yet, compared to their Pb-based counterparts, Sn-based perovskites so far show inferior device performance, and they are even more sensitive to degradation because of self-oxidation^19^. In addition, Sn is also enlisted as a harmful chemical, raising concerns regarding its suitability as a more environmentally friendly alternative to lead in perovskite solar cells^29^,^30^.

In this paper, we take the first step in comparing the environmental impact of Pb and Sn-based perovskite solar cells. One important scenario to be addressed could be a structural failure of a solar panel, resulting in (i) the full degradation of its perovskite absorber material when being exposed to ambient, followed by (ii) the dissolution of the decomposition products in water on a rainy day later on. Thus, as a first step, the main degradation products of Pb and Sn-based perovskites need to be established before proceeding to their toxicological assessment in an aqueous environment by means of the model organism zebrafish *Danio rerio*, employing the Zebrafish embryo acute toxicity testing (ZFET) protocol. Several characteristics of embryo and/or larvae indicate that these non-mammalian models can bridge the gap between the simple *in vitro* studies (cell culture assays), and biological validation in whole animal models (mammals)^31^. The choice for zebrafish as model organism is also based on the availability of well-established and specific test procedures and protocols (provided by the Organization for Economic Co-operation and Development (OECD))^32^,^33^. Although the zebrafish cannot replace rodent models completely, the accepted protocols create a “gold standard” that enables to create a large throughput of samples and also obtain test results fast and efficiently, contrary to analogue experiments on mice or rats^31^,^32^. The larvae being transparent and consistent in size allows for easy observation of the fully genome-sequenced zebrafish (with the optional use of staining methods), so they can serve as a sensitive model to study developing pathologies and genotoxicity. The tests are conducted during the first 4 days of the fast embryonic development of these fish, and the obtained toxicological results are directly transferable to the human genetics for 85%^34^. The results from our study therefore lead to a first nuanced comparison of the environmental impact of degraded Pb and Sn-based perovskites.

## Results and Discussion

**Figure 1:**
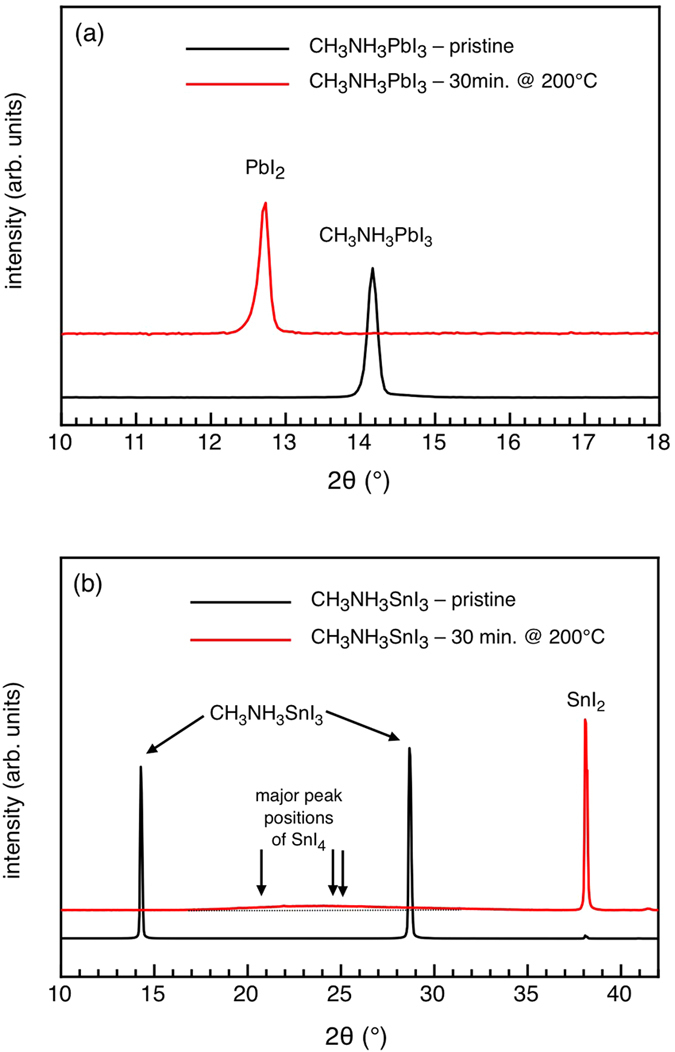
X-ray Diffractograms of (a) pristine and degraded CH_3_NH_3_PbI_3_ and (b) pristine and degraded CH_3_NH_3_SnI_3._

The chemical degradation pathways for the CH_3_NH_3_PbI_3_ perovskite were studied before by others and our-selves^14^,^16^,^23^,^24^,^35^,^36^. The general consensus is that mild humidity leads to a reversible hydrated perovskite-H_2_O complex^23^,^24^,^35^, and upon complete disintegration of the perovskite crystal, PbI_2_ and CH_3_NH_3_I are formed, which can in part further degrade into CH_3_NH_2_, HI, and I_2_, in parallel with Pb clusters being formed, but only to a lesser extent^14^,^16^,^36^. There are no published studies on the degradation of CH_3_NH_3_SnI_3_, but the focus of this work makes an elementary comparison between degraded Pb-based and Sn-based perovskites apposite using X-Ray Diffraction (XRD) as material-sensitive probe, it can be recognized in Fig. 1a that annealing of a pristine CH_3_NH_3_PbI_3_ film for 30 min. at 200 °C in air induces, as expected, the complete transformation of the perovskite into PbI_2_^14^,^16^,^36^. In close analogy, the same heat treatment of CH_3_NH_3_SnI_3_ is found to fully degrade the pristine film^25^ into SnI_2_ with a certain admixture of SnI_4_ as suggested by a rather broad peak centered at around 23° (see Fig. 1b)^37^. Using the Scherrer formula for a rough estimate of the corresponding crystal size, a value of 1–3 nm can be derived, pointing to the presence of small SnI_4_ nanoclusters in the degradation product (which will be neglected in the following). The predominant presence of SnI_2_ is confirmed by X-ray Photoelectron Spectroscopy (XPS) measurement (see Fig. S1), revealing one single spectral component at the binding energy that corresponds to the expected value for SnI_2_.

*Ergo*, PbI_2_ and SnI_2_ are the main metal containing degradation compounds, specific to the corresponding Pb and Sn-based perovskite solar cells, that need to be investigated in the light of their potential environmental burden. To guarantee consistent purity and avoid possible synergistic effects with scantily present metallic clusters, the commercial versions of these chemicals were used as test subjects for our systematic study. As such, relevant quantities of PbI_2_ and SnI_2_ were added to the aquatic environment of zebrafish *Danio rerio* embryos (simulated by E3 medium, a dilute salt solution, see Methods) to examine their respective influence on these organisms, following procedures according to the “OECD guidelines for the testing of chemicals 236”, using the ZFET protocol^32^,^33^. Herein, lethality and effect occurrence (defective phenotypes) in the embryos are monitored at specific points in time.

**Figure 2:**
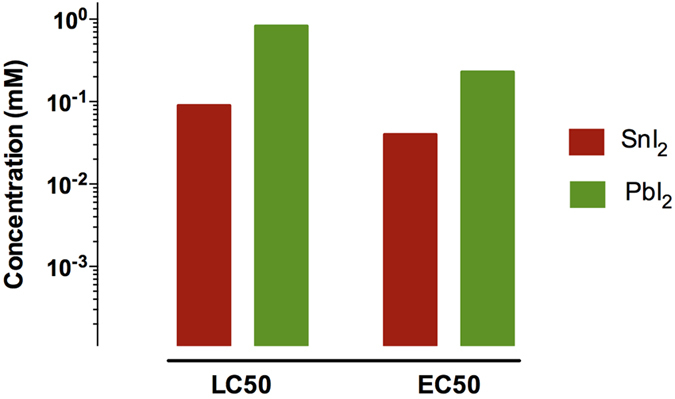
Schematic overview of LC50 and EC50 values of tested compounds at 4 dpf, based on nominal concentrations of SnI_2_ and PbI_2_.

To provide a first overall picture of the toxicity of both toxins on a molar equivalent level, the LC50 (Lethal Concentration for 50% of the population) and EC50 (Effect Concentration occurring for 50% of the population) values at the end-point measure of 4 days post fertilization (dpf) were determined from the obtained concentration-survival and concentration-malformation curves (*vide infra*), and are shown in Fig. 2. With an LC50_4dpf_ of 0.09 mM nominal concentration, SnI_2_ has a much lower lethality onset than PbI_2_ (0.83 mM nominal concentration). This means that exposure to SnI_2_ is more lethal than PbI_2_ at the same (nominal!) concentrations, but for different reasons, as will become clear in the remainder of this paper. Correspondingly, the same trend was found for the EC50_4dpf_, having a value of 0.04 mM nominal concentration for SnI_2_ and 0.23 mM nominal concentration for PbI_2_, inferring that effects observed upon SnI_2_ exposure have an inception at lower concentrations compared to the PbI_2_ counterpart. Even without further investigation, these findings acutely challenge the widespread view of the supposed environmentally friendly character of Sn-based perovskites compared to Pb-based ones.

**Figure 3:**
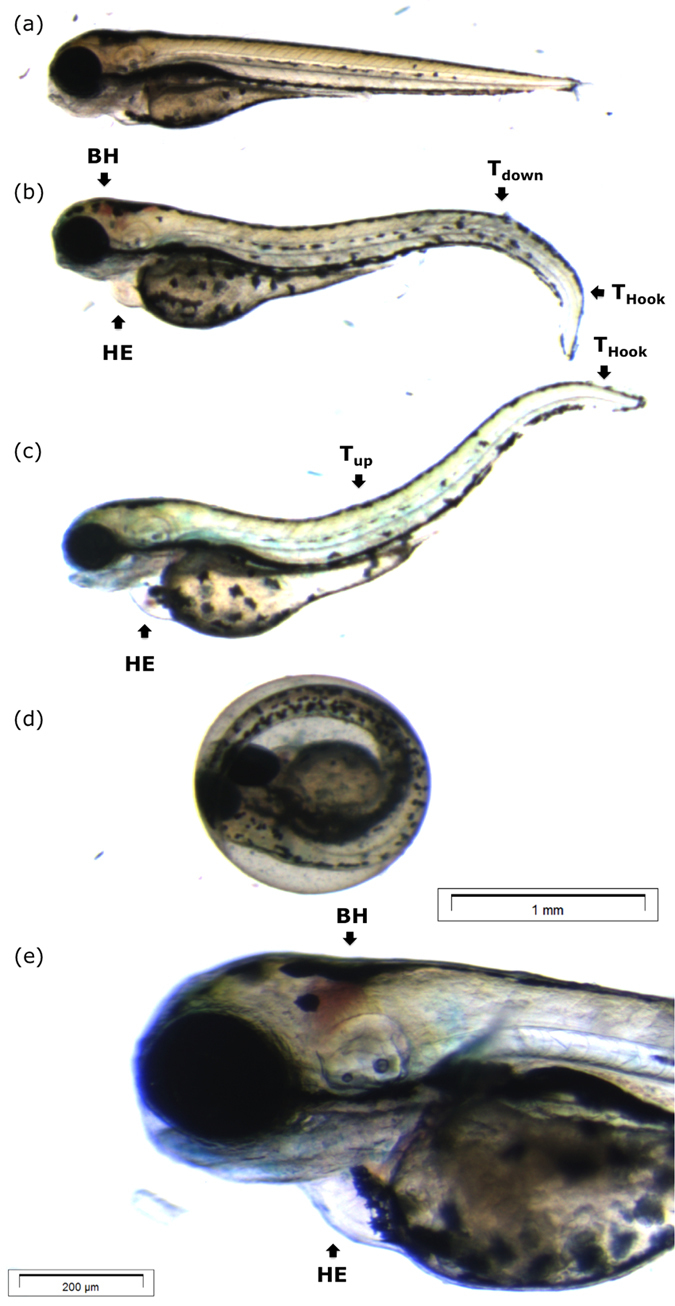
Morphological defect phenotypes 4 dpf. (**a**) Control larva with straight trunk, no aberration in heart and brain region, pigmented and clearly segmented body. (**b**) Larva observed with mixed defect: dorsal curvature tail down (T_down_) and hooked tail (T_hook_), heart oedema (HE) and brain haemovascular defect (BH). (**c**) Larva observed with mixed defect: dorsal curvature tail up and hooked tail, and heart oedema. (**d**) Un-hatched larva (**e**) magnification of (**b**) illustrating a brain haemovascular defect in detail. Scale bars: (**a–d**): 1 mm; (**e**): 200 μm.

**Figure 4:**
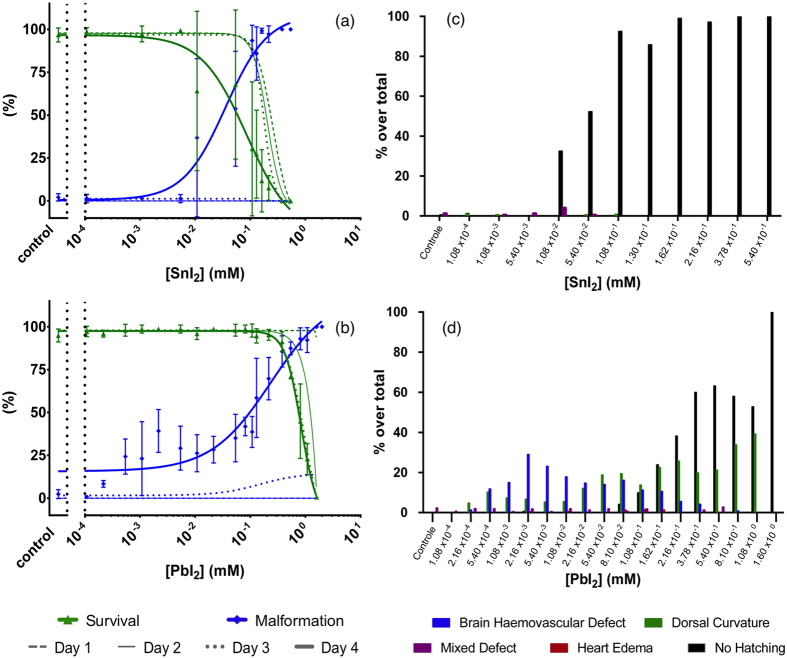
Nominal concentration-survival curve (green), concentration-malformation curve (blue) for SnI_2_ (**a**) and PbI_2_ (**b**) treated embryos. For clarity, only the malformation and survival data at 4 dpf are presented as mean ± SD, while 1 dpf, 2 dpf and 3 dpf are depicted by lines without bullets or error bars. (**c,d**) represent the prevalence of morphological defect phenotypes at 4 dpf for SnI_2_ and PbI_2_, respectively.

On a more profound level, the occurrence of various individual morphological defects upon intoxication was examined with respect to the normal embryonic stages of development as reported by Kimmel *et al.*, as well as the developmental defects upon exposure to heavy metals as reported by Jezierska *et al.*^38^,^39^ Besides the defect of “no hatching”, they comprise: heart oedema (HE), brain haemovascular defect (BH), abnormal trunk: upward bent trunk (T_up_), downward bent trunk (T_down_) and hooked tail (T_hook_). These defects are illustrated in Fig. 3, and are described in more detail in the Supplementary Information. We now address the observed individual prevalence of these defective phenotypes (collectively referred to as “malformations”) as a function of SnI_2_ and PbI_2_ concentration in the growth medium, in conjunction with the corresponding concentration-survival and concentration-malformation records. Figure 4a shows that, for the case of SnI_2_, 100% lethality occurred already at 1 dpf for the highest tested concentration of 0.54 mM. In the following days, also for lower concentrations the mortality increased. Apart from a scarce amount of mixed instances, non-hatching was the only observed defective phenotype and was found to be dose-dependent (see bar chart in (Fig. 4c)). In stark contrast, in the case of PbI_2_ treated embryos, 100% lethality occurred only by 2–3 dpf at the highest concentration of 1.60 mM (Fig. 4b). Remarkably, as compared to the SnI_2_ exposure, more defect phenotypes were observed: at first sight, the concentration-response curve suggests near dose-dependent malformation, however, when the defect phenotypes are considered separately (as illustrated in the bar chart in Fig. 4d), a multi-phasic appearance of defect phenotypes is seen. The dorsal curvature is the only defect with a discernable dose-response trend (with T_up_ and T_down_ being increasingly accompanied by T_hook_ for higher concentrations, and simultaneously also the trunk is more drastically curved (not shown individually in the graph)). The BH defect is dose-dependent at lower concentrations but then fades away at higher concentrations where the non-hatching phenotype –as previously found in SnI_2_-treated embryos– becomes prevalent.

**Figure 5:**
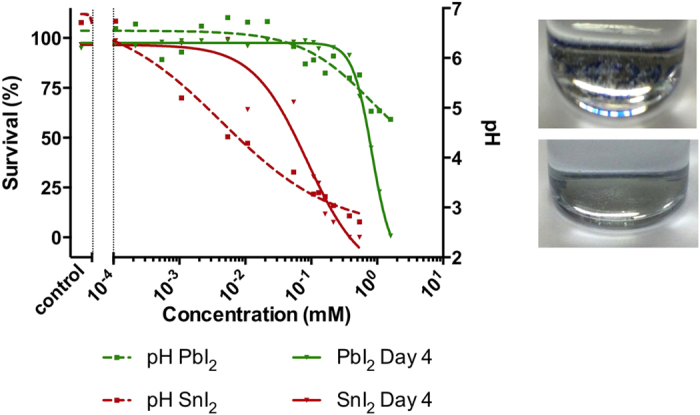
Left: pH (broken lines) and lethality record (full lines) in function of nominal concentration. Right: Precipitation in SnI_2_ (top) and PbI_2_ (bottom) long-term stored stock solutions (~3 weeks).

Notwithstanding the straightforward finding of higher toxicity upon SnI_2_ exposure compared to PbI_2_, the dissimilar expressions of their resulting intoxication suggest that different mechanisms are involved. Particularly, the prevalence of non-hatching embryos for a large range of concentrations, an observation much more distinct for SnI_2_ than for PbI_2_, points to a retarded embryonic development, which is –aside from exposure to heavy metals– also often linked to the acidity of the growth medium^39^,^40^,^41^. Taking this into consideration, the pH of the E3 medium was measured as a function of concentration of either toxin. Figure 5 shows these pH measurements (broken lines), together with the lethality record at 4 dpf (full lines). It is immediately apparent that a relation exists between the pH of the medium and the concentration of the added toxin. In case of PbI_2_, the pH is quite constant at values around 6.5 up to a concentration of 0.1 mM, and then decreases to a value around 5 for the highest concentration. Contrarily, the slightest addition of SnI_2_ has a prompt impact on the pH and acidifies the medium over the whole range of concentrations to a pH below 3 for the highest one. Overlaying now the pH data with the lethality records reveals that they show the same trend, suggesting that the acidity of the medium upon SnI_2_ or PbI_2_ addition is at least in part responsible for the impaired development of the embryos, besides the expected heavy metal intoxication^39^,^41^,^42^,^43^,^44^,^45^.

**Figure 6:**
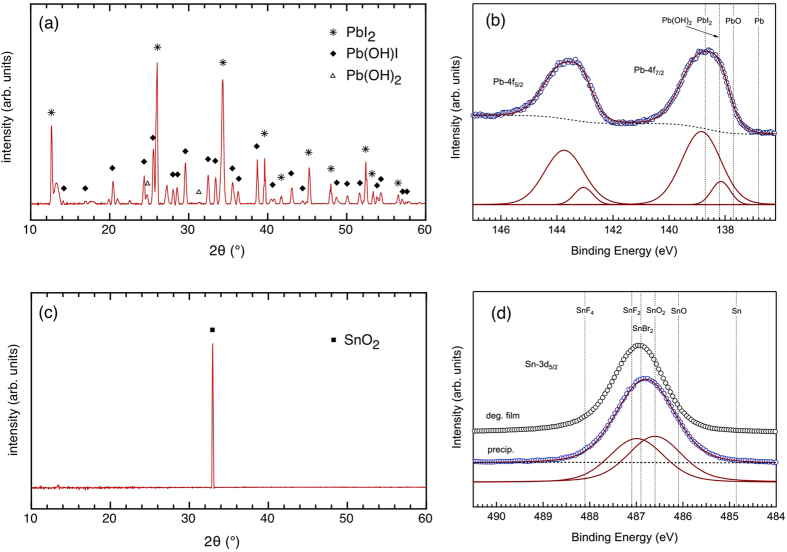
(**a**) X-ray diffractogram of the precipitate in the PbI_2_ solution and (**b**) corresponding high-resolution XPS core level scan of the Pb-4f binding energy region, together with the deconvolution into single components. (**c**) X-ray diffractogram of the precipitate in the SnI_2_ solution and (**d**) corresponding high-resolution XPS core level scan of the Sn-3d binding energy region (blue circles), together with the deconvolution into single components. The spectrum of a degraded Sn-based perovskite film (representing SnI_2_) is added as a comparison (black circles). Binding energy reference values can be found in Table S1. Note that there is no binding energy for Pb(OH)I available but it will likely coincide with that of Pb(OH)_2_.

To understand these effects better, we turn our attention to the likely origin of the observed acidity in the solutions with added SnI_2_ and PbI_2_. While at first, it was suspected that the drop in pH upon increasing toxin concentration simply resulted from the absence of a buffer in the E3 medium (which is a condition chosen purposefully to simulate the most likely scenario of intoxication, as it would happen in nature), the strong dose-dependent reduction in pH, as shown in Fig. 5, suggests that there are underlying chemical variables. Zhu *et al.* and Dennis *et al.* have shown that pH-induced partial conversions from PbI_2_ to leadhydroxyiodide (Pb(OH)I) and/or leadhydroxide (Pb(OH)_2_) can occur^46^,^47^,^48^. This effect does indeed seem to appear, as evidenced by Fig. 6a, showing XRD data of a very small amount of precipitate that could be collected from the highest concentrated solution of PbI_2_, proving that Pb(OH)I is formed, together with a trace amount of Pb(OH)_2_^47^,^49^. Corresponding XPS data confirm that the Pb from PbI_2_ is not the only Pb component present (see Fig. 6b). Interestingly, in the work of Zhu *et al.* it has also been shown that such conversion is efficient at neutral pH, and continues in a self-maintaining manner, suggesting the formation of hydroiodic acid (HI) until pH 4 is attained^46^,^47^. This correlates very well with the observations in Fig. 5, where in fact the concentration-dependent decrease in pH does not undershoot a value of 4 in case of PbI_2_. Based on these arguments, the proposed reactions for PbI_2_ are:


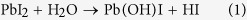



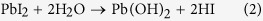


An analogous study was performed on the readily formed precipitate in the SnI_2_ containing medium. The XRD measurement in Fig. 6c indicates that the precipitate contains a large portion of SnO_2_^50^. This is corroborated by XPS measurement of the corresponding Sn-3d core level, which now comprises two components: one at lower binding energy that indeed coincides with the expected value for SnO_2_ (486.6 eV) and one at higher binding energy that agrees with SnI_2_ as detected in a degraded Sn-based perovskite film (486.95 eV, see also Fig. S1). The fact that the significant contribution of SnI_2_ in the XPS spectrum does not show up in the XRD measurement might be attributed to the presence of an amorphous phase or of very small nanoclusters (size ≤ 1 nm) resulting in extremely broad (and thus hard to detect) diffraction patterns according to the Scherrer formula. Based on these results, and taking into account the self-oxidation of Sn^2+^ to Sn^4+^ in ambient atmosphere^19^, the proposed reaction for SnI_2_ in aqueous medium is:


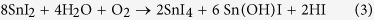


Where the observed presence of SnO_2_ suggests that the formed SnI_4_ could react further as:


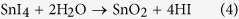


Just like in case of PbI_2_, the accumulation of HI in the several possible decomposition pathways again correlates with the low pH values measured in the medium. Since the Sn(OH)I was not explicitly detected we hypothesize that its binding energy coincides with that of SnO_2_. As Sn(OH)I is prone to self-oxidation (because of the divalent nature of the Sn ion, in the same way as for SnI_2_), it is also conceivable that it was present initially but then quickly oxidized.

**Figure 7:**
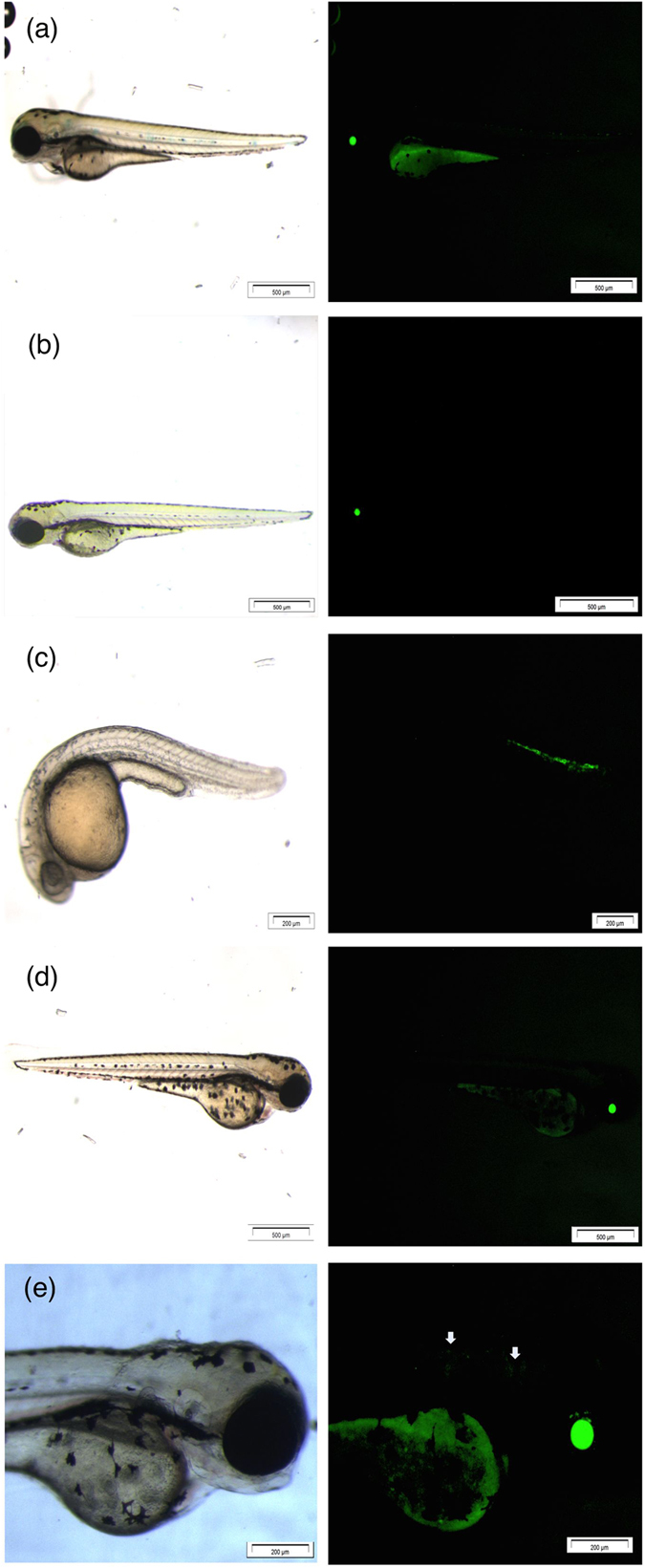
Stereoscopic (left-hand side) and fluorescent pictures (right-hand side) of Tg(hsp70l:GFP) with (**a**) control, (**b**) SnI_2_ 3 dpf at 2.16·10-1 mM, (**c**) PbI_2_ 1 dpf at 1.08 mM, (**d**) PbI_2_ 3 dpf at 5.4·10-3 mM and (e) being the magnification of (**d**). Scale bars: (**a**): 500 μm; (**b**): 500 μm; (**c**): 200 μm; (**d**): 500 μm; (**e**): 200 μm.

It was reproducibly observed that the more acidic Sn containing medium showed a much larger amount of precipitate in comparison with the more neutral Pb containing medium (see Fig. 5, right-hand side). As discussed above, part of the metal content resides in the precipitates formed over time and, depending on the severity of precipitation, this can therefore lead to a weakened intoxication of the embryos by the heavy metals. Accordingly, the actual Pb and Sn content of the E3 medium was examined. A close estimate was provided by performing XPS on a dried droplet of E3 medium containing either 1.08 mM of PbI_2_ or SnI_2_, using the known concentration of Ca and Mg salts in the E3 medium as internal reference. Estimates based on Fig. S2 reveal that the actual concentration for an added 1.08 mM SnI_2_ is only 8 μM, and thus most of the Sn ends up in the readily formed precipitate. This means that the embryos are exposed to only a small fraction of the added Sn concentration, and thereby strongly suggests that their decelerated rate of development, expressed by the non-hatching defective phenotype, is much more a pH-induced effect rather than a result of exposure to Sn as heavy metal. This is also strongly supported by the work of Johanssen *et al.*, who have demonstrated the detrimental consequences of low pH on the development of zebrafish^51^, and such effect has even been demonstrated for a variety of aquatic organisms (frogs, salmon, minnow, catfish, pikes)^52^,^53^,^54^,^55^,^56^,^57^. In contrast, the actual concentration for an added 1.08 mM of PbI_2_ amounts to 1 mM, which is close to being fully dissolved, with only a small quantity being comprised in the precipitate over time. Accordingly, in case of PbI_2_ a combined effect of the pH and the heavy metal itself in fact are inflicted upon the embryos. This aptly clarifies why complex appearances of multiple defect phenotypes were observed in case of PbI_2_: Fig. 5 illustrates that, on the one hand, the reduced pH becomes most pronounced at higher exposure concentrations, such that it corresponds to the dose-dependent increase of delay-induced non-hatched phenotypes. On the other hand, embryos that did manage to hatch at these lower pH values still show a dose-response increase of the dorsal curvature, which is clearly a result of heavy metal intoxication^43^. The BH defect diminishes for lower pH, but nevertheless also indicates interference during development resulting from intoxication. Although the latter is not completely understood, it does not dispel the general conclusion that in simulating the most realistic scenario of intoxication (minimal or no buffering involved as it would be in nature), the SnI_2_ has a larger burden on the environment by means of virtually solely reduced pH, when compared to PbI_2_, which contributes by means of a combined pH-heavy metal effect. To substantiate this, complementary analysis by means of Heat Shock Protein 70l:Green Fluorescent Protein (hsp70l:GFP) fluorescence in transgenic zebrafish was performed. In such fish, a GFP protein emits green fluorescent light upon expression of the stress-activated hsp70 gene. Since not all stress-induced effects by PbI_2_ and SnI_2_ exposure in the embryos are directly visually accessible, such fluorescent analysis can provide additional phenotypic information that otherwise remains unrevealed^58^,^59^. Figure 7 shows stereoscopic (left-hand side) and fluorescent (right-hand side) pictures of the transgenic embryos exposed to the relevant chemicals. The fluorescence signal from the control embryo (Fig. 7a), observed in the yolk region and in the eye lens is in fact auto-fluorescence, due to basal activity of the *hsp70l* promoter. Both are a sign of normal embryonic development^60^. A deviation from these characteristics can be seen when comparing this signal to the embryo exposed to SnI_2_ (Fig. 7b), where a dim fluorescent signal in the eye region and complete absence of fluorescence in the yolk region are detected. Thus, even though the stereoscopic image appears normal, the dim fluorescence is a tell-tale sign of impaired embryonic development, which aligns with our findings as described in the above^60^. Additional fluorescence was not at all observed upon exposure to SnI_2_. This again confirms the rationale that the majority of the heavy metal is collected in the precipitate as Sn(OH)I or SnO_2_, and the residual Sn ions actually dissolved in the test solution are not contributing to defective phenotyped embryos.

The transgenic embryos exposed to PbI_2_ are shown in Fig. 7c,d. They exhibit two distinct morphological defects as described before: dorsal curvature T_down_ recorded at 1 dpf (Fig. 7c) and brain haemovascular defect recorded at 3 dpf (Fig. 7d), as is apparent from the stereoscopic images. The fluorescent images of both defect phenotypes correspond well with the stereoscopic images, showing additional fluorescence in the bent region of the trunk and dim fluorescence in the head and neck region (Fig. 7e, magnified and pinpointed by arrows). Contrary to the SnI_2_ case, no differences in terms of reduced auto-fluorescence could be distinguished that could potentially further relate to the non-hatched defect phenotype from the ZFET observations, due to a delayed development. Lastly, also no additional fluorescence indicating damage to other tissues, which would not have been distinguishable stereoscopically, was observed. Altogether, these observations strengthen the previous findings on SnI_2_ exposure being more toxic than PbI_2_, not through exposure to the heavy metal itself but through the acidic pH resulting from SnI_2_ decomposition in ambient atmosphere. PbI_2_ exposure leads to a mixed effect as both phenotypes appear: dorsal curvature and brain haemovascular defect (Pb intoxication), as well as non-hatching embryos at 4 dpf (developmental delay).

Finally, to put our results into a more general perspective, we provide a numerical example that illustrates the intoxication hazard associated with Pb- and Sn-based perovskites in a practical situation. Consider a one square meter CH_3_NH_3_PbI_3_ perovskite module on a rooftop that is shattered due to fierce, penetrative hail, and the resulting degradation product PbI_2_ running down a drainpipe into a garden under rainy conditions subsequently. Assuming such a module contains 0.9 g of PbI_2_^17^, the full dissolution of this amount into about 2.5 liters of water would result in the LC50 value obtained from our study. Approximately 20 liters would be required to dilute the PbI_2_ to a concentration that would not be lethal to zebrafish, and roughly 20'000 liters would be needed to obtain a harmless dilution, inducing no defects. In case of Sn-based panels even more water would be needed to dilute to a non-deadly concentration (270 liters), and 270'000 liters for a harmless concentration. On the other hand, assuming heavy rainfall, only 4–10 liters of water per m^2^ per hour would be available for dilution, which now allows to better estimate the magnitude of the problem. Given the 85% transferability of the toxicological results from zebrafish to humans, this example clearly illustrates that while on a global scale the danger of heavy metal intoxication from faulty perovskite modules might be negligible, locally a significant burden on the environment may exist.

## Conclusion

In conclusion, the environmental impact of PbI_2_ and SnI_2_, representing the main degradation products of the corresponding organometal halide perovskite solar cells, was tested by means of the model organism zebrafish *Danio rerio* according to the ZFET. At equal concentration for both compounds, a higher lethal response was found in the embryos exposed to SnI_2_ than for PbI_2_. Furthermore, it could be revealed that lethality rates and morphological defects of SnI_2_ treated embryos are not due to the presence of the heavy metal, but rather due to reduced pH value. This is explained by the fact that SnI_2_ is a highly unstable product in ambient atmosphere, causing immediate decomposition into HI (the acidifier), along with toxicologically inactive oxygenated Sn precipitates. Upon exposure to the Pb counterpart, a combined effect of the heavy metal itself along with the reduced pH is observed, as evidenced by the complex multi-phasic appearance of the morphological record, where the presence of mainly Pb(OH)I precipitate was found, along with smaller amounts of HI. Since both Sn and Pb are present here as inorganic compounds, it must be noted that their uptake is much lower in comparison to analogous organic molecules (as in, for example, tetrabutyllead in leaded gasoline or tributyltins in industrial biocides); Nevertheless, the release of HI introduces a new perspective in the possible routes of toxicity. Notably, the strong acidification induced by the SnI_2_ is found to be more harmful than the combined effect of milder acidification and the expected Pb intoxication induced by PbI_2_. These results challenge the current view in solar cell development, where Sn-based perovskites are regularly put forward as the non-toxic alternative of Pb-based ones. This leads to the recommendation that the enthusiasm towards the purported environmental friendliness of Sn-based perovskites compared to Pb-based ones should be approached with great caution.

## Methods

The Zebrafish embryo acute toxicity testing (ZFET) protocol was employed, provided by the Organization for Economic Co-operation and Development (OECD). For each compound, 3 independent replicate experiments were performed, with each replicate having n = ± 50 embryos per tested condition. The ZFET array represents a rapid, reliable and inter-laboratorial comparable route for acute toxicity screening, as highly recommended by the European Union Reference Laboratory for Alternatives to Animal Testing (EURL-ECVAM) in 2014 (peer reviewed by the European Scientific Advisory Committee)^32^. Zebrafish wild type strain AB (ZIRC, USA) and transgenic fluorescent strains Tg(hsp70l:GFP) were maintained within the zebrafish facility in GIGA-Research, University of Liège (licence LA2610359). The fish are reared in a Techniplast recirculating system under 14:10 hour light/dark photocycle. For breeding, a stock of non-treated mature zebrafish is used for egg production. 12 hours day/night regime prior to the breeding, males and females were kept at a ratio 1:2 in a breeding chamber with a separator to prevent undesired spawning. The breeding procedure was continued the next day by removing the separating slits of the breeding tanks, after which the fish were placed in fresh system water. To prevent cannibalism of the eggs by the adult zebrafish, spawn traps are foreseen at the bottom of the breeding tanks. Spawning took place in the following 30 to 60 minutes, after which the eggs were collected and placed into E3 medium (5 mM NaCl, 0.17 mM KCl, 0.4 mM CaCl_2_ and 0.16 mM MgSO_4_ in H_2_O, pH 6.7). The point of spawning, i.e. the removal of the separation slit, was marked as zero hours-post (hpf) fertilisation. The breeding date was also marked as zero days post fertilization (dpf). After a 30 minute incubation at 28 °C, the eggs are sorted by means of a stereomicroscope. A synchronous cleavage was constrained and eggs following this cleavage pattern were identified as clearly fertilized and normal (≥80%). The sorting procedure could continue until 3 to 4 hpf and was finished by mixing eggs from high-fertile pools and distributing them in 24 well-plates, with 6 to 7 embryos per well. Thereafter, the chemical exposure was started, for which the E3 medium in each well was replaced with the test solutions. Less than 0.3 vol% DMSO was added to the stock solutions for both toxins to account for their limited solubility, which is well within the acceptable range to avoid any additional impact on the embryos^33^,^61^. The plates were incubated at 28 °C and were renewed daily until 4 dpf in order to keep the exposed concentration maximized as well as to remove metabolic waste and renew dissolved oxygen^2^. Embryonic endpoints were established by comparing phenotypes with those detailed by Kimmel and co-workers^38^. The observations were performed with a stereomicroscope; larvae were photographed using an SZX10 stereomicroscope coupled to an XC50 camera (Olympus). From these experiments, data was calculated to determine indices such as median lethal concentration (LC50) and median effective concentration (EC50), using non-linear 4-parameter regression in the Prism software package (version 6). For the Tg(hsp70l:GFP) strain, the same protocol of maintenance, breeding and testing was employed as in ZFET. The observations were altered to 1 and 3 dpf to maximize the fluorescent signal. All protocols for experiments were evaluated by the Institutional Animal Care and Use Committee of the University of Liège and approved under the file number 13-1506 (licence LA 1610002). The methods were carried out in accordance with the approved guidelines.

XRD measurements were performed with a Bruker D8 diffractometer with Cu Kα1 radiation. Chemical analysis was carried out on a commercial electron spectrometer (PHI-5600LS) with an X-ray source providing monochromatized Al-Kα photons (1486.6 eV). Core level spectra were acquired setting the total energy resolution of the spectrometer (photons and electrons) to 0.36 eV full width at half maximum (FWHM). Experimental data were fitted following the procedure introduced by Wertheim *et al.*^62^

## Additional Information

**How to cite this article**: Babayigit, A. *et al.* Assessing the toxicity of Pb- and Sn-based perovskite solar cells in model organism *Danio rerio*. *Sci. Rep.* **6**, 18721; doi: 10.1038/srep18721 (2016).

## Supplementary Material

Supplementary Information
