# Supplementary material for: Assessing the toxicity of Pb- and Sn-based perovskite solar cells in model organism Danio rerio
Source: Sci Rep. 2016 Jan 13;6:18721. doi: 10.1038/srep18721 (PMC4725943; doi:10.1038/srep18721)
Supplement: Supplementary Information [file srep18721-s1.doc]

**Supplementary Information for:**

**Assessing the toxicity of Pb- and Sn-based perovskite solar cells in model organism *Danio rerio***

Aslihan Babayigit^1^, Dinh Duy Thanh^2^, Anitha Ethirajan^1^, Jean Manca^3^, Marc Muller^2^, Hans-Gerd Boyen^1^, Bert Conings^1^

^1^ Hasselt University, Institute for Materials Research, Wetenschapspark 1, 3590 Diepenbeek, Belgium

^2^ University of Liège, Laboratory for Organogenesis and Regeneration, GIGA-Research, B34, Avenue de l'Hôpital 1, 4000 Sart-Tilman, Belgium

^3^ Hasselt University, X-LaB, Agoralaan 1, Building D, 3590 Diepenbeek, Belgium

**Clarification of the morphological defects depicted in Figure 2.**

(i) No hatching: During normal development larvae hatch around 72 hours post‑fertilization (hpf) from the chorion. When this was not observed at 4 days post‑fertilization (dpf), the larvae are considered deviant from normal embryonic development.

(ii) Heart oedema (HE): Abnormal accumulation of fluids inside the embryo’s heart cause the skin around the heart to swell, resulting in pressure to internal structures, even so that abnormal shaping can be resultant.

(iii) Brain haemovascular defect (BH): There are two phenotypes, both appearing as red spots due to local blood accumulation. Haemostasis is the accumulation of blood inside the cardiovascular system obstructed by thrombosis or stenosis. Haemorrhage is internal bleeding which causes blood to accumulate outside of the cardiovascular system.

(iv) Abnormal trunk: During normal development, the trunk of the embryo has minimal curvature at 4 dpf, but exposure to chemicals can induce abnormal bending of the trunk. For this phenotype, two distinct variations are observed. The first one is described as a dorsal curvature in which the tail can be directed up- or downwards with respect to the yolk (T_up_; T_down_). The second bending is the same dorsal curvature, but accompanied by a hooked tail (T_hook_) that in itself can bend both up- and downwards as well, originating from the most posterior part of the tail.

(v) Combined defect: Dorsal curvature, heart oedema and/or brain haemovascular defect in the same animal.


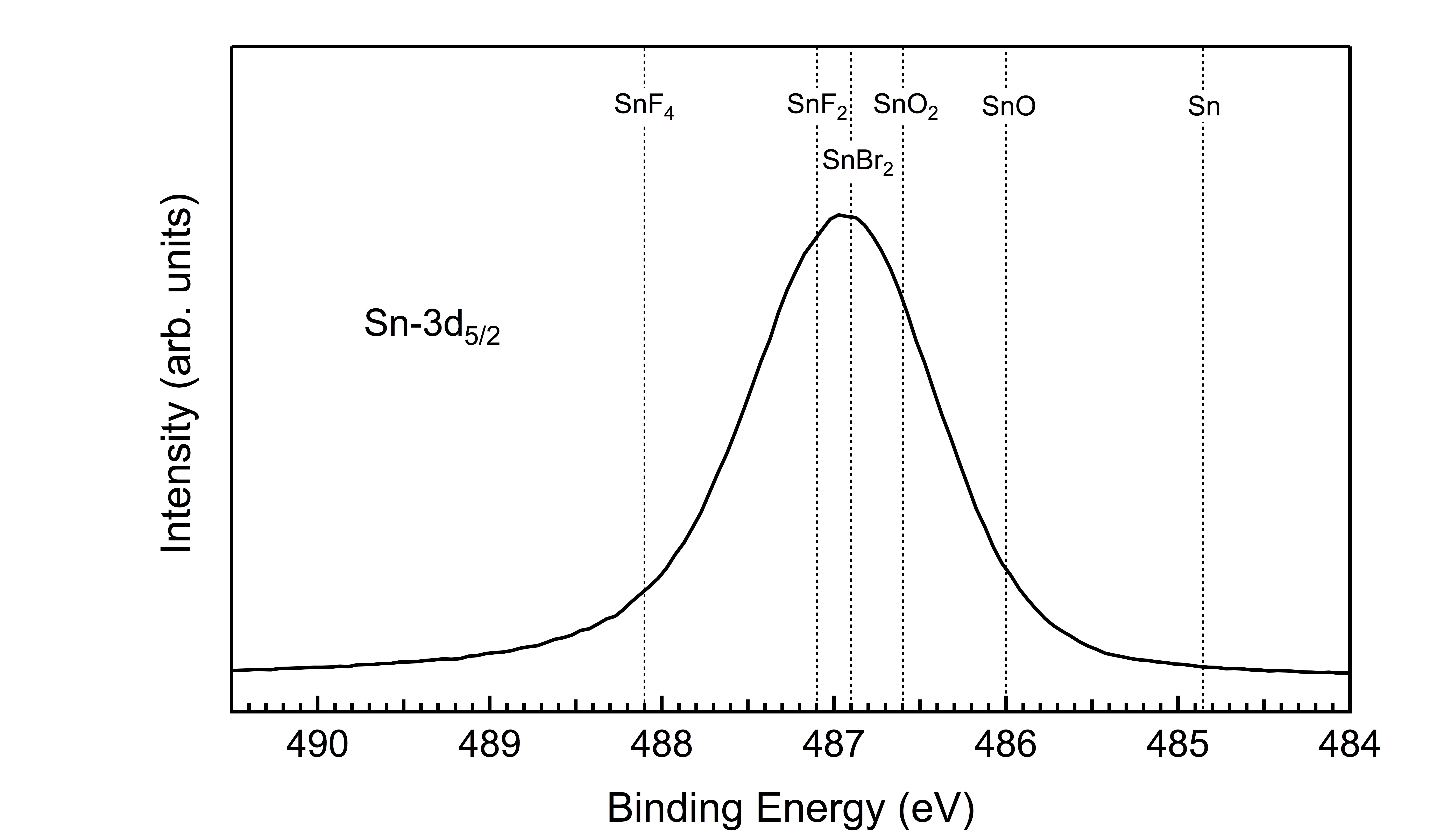


**Figure S1:** High resolution XPS core level scan in the Sn-3d_5/2_ binding energy region, of a degraded CH_3_NH_3_SnI_3_ perovskite film. The CH_3_NH_3_SnI_3_ film was prepared following a slightly modified procedure as described in Noel, N. K.*, et al.* Lead-free organic-inorganic tin halide perovskites for photovoltaic applications. *Energy Environ. Sci.* 7, 3061-3068 (2014).


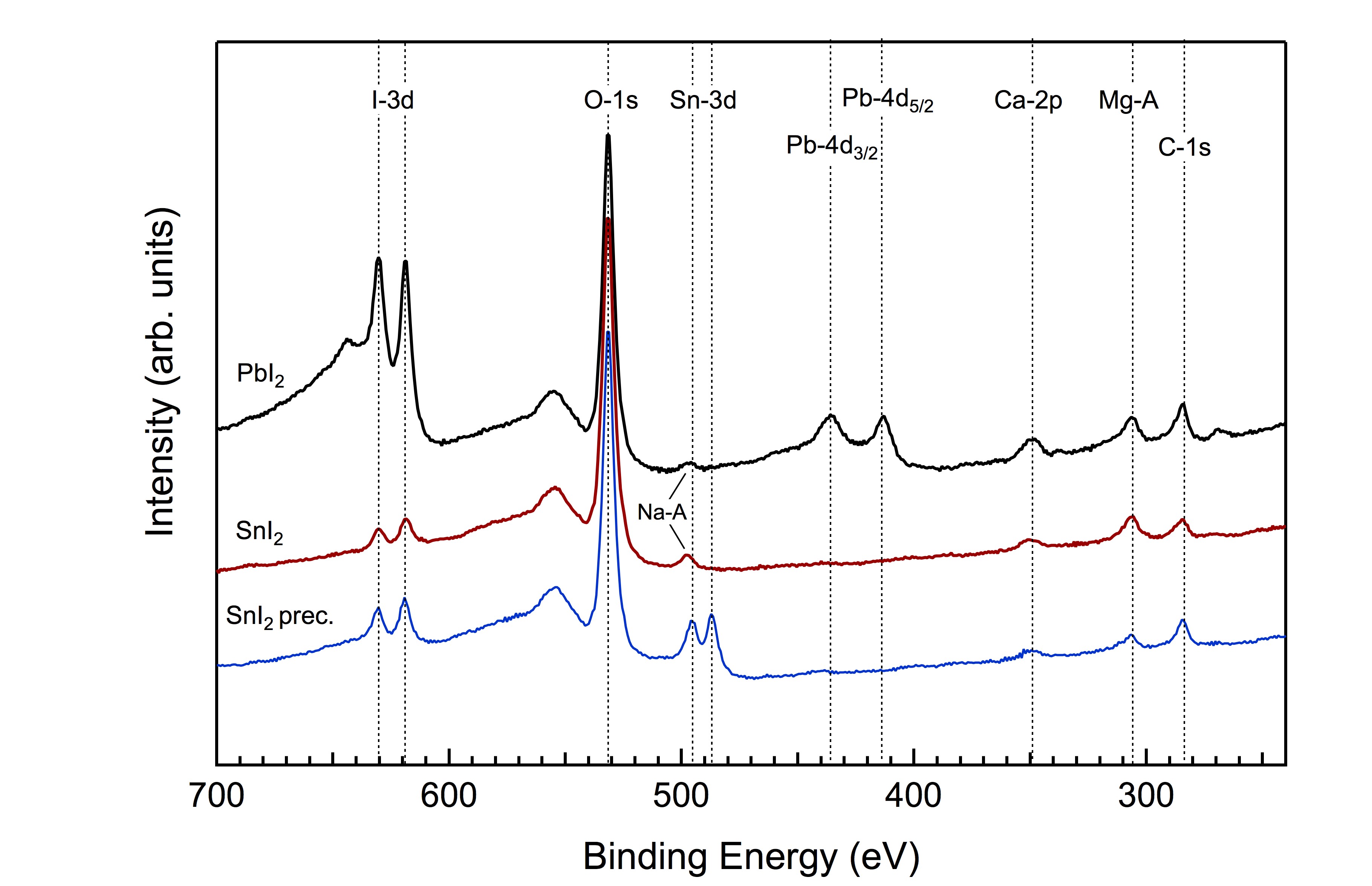


**Figure S2:** XPS measurements of PbI_2_ stock solution, SnI_2_ stock solution and SnI_2_ precipitate.

**Table S1:** Reference binding energy values

| **core level** | **chemical** | **binding energy value (eV)** | **reference** |
| --- | --- | --- | --- |
| Pb-4f_7/2_ | Pb | 136.8 | 1 |
| Pb-4f_7/2_ | PbO | 137.7 | 2 |
| Pb-4f_7/2_ | Pb(OH)_2_ | 138.2 | 1, 3 |
| Pb-4f_7/2_ | PbI_2_ | 138.7 | 4 |
| Sn-3d_5/2_ | Sn | 484.85 | 5 |
| Sn-3d_5/2_ | SnO | 486.1 | 6 |
| Sn-3d_5/2_ | SnO_2_ | 486.6 | 7 |
| Sn-3d_5/2_ | SnBr_2_ | 486.9 | 8 |
| Sn-3d_5/2_ | SnF_2_ | 487.1 | 8 |
| Sn-3d_5/2_ | SnF_4_ | 488.2 | 9 |

**References**

1. Pederson, L. R. Two-dimensional chemical-state plot for lead using XPS. *J. Electron. Spectrosc. Relat. Phenom.* **28**, 203-209 (1982).

2. Zingg, D. S. & Hercules, D. M. Electron spectroscopy for chemical analysis studies of lead sulfide oxidation. *J. Phys. Chem.* **82**, 1992-1995 (1978).

3. Nefedov, V. I., Salyn, Y. V., Solozhenkin, P. M. & Pulatov, G. Y. X-ray photoelectron study of surface compounds formed during flotation of minerals. *Surf. Interface Anal.* **2**, 170-172 (1980).

4. Morgan, W. E. & Van Wazer, J. R. Binding energy shifts in the x-ray photoelectron spectra of a series of related Group IVa compounds. *J. Phys. Chem.* **77**, 964-969 (1973).

5. Wagner, C. D.*, et al.* Handbook of X-Ray Photoelectron Spectroscopy. *Perkin-Elmer Corporation, Physical Electronics Division, Eden Prairie, Minn. 55344* (1979).

6. Stranick, M. A. & Moskwa, A. SnO by XPS. *Surf. Sci. Spectra* **2**, 45-49 (1993).

7. Stranick, M. A. & Moskwa, A. SnO2 by XPS. *Surf. Sci. Spectra* **2**, 50-54 (1993).

8. Grutsch, P. A., Zeller, M. V. & Fehlner, T. P. Photoelectron spectroscopy of tin compounds. *Inorg. Chem.* **12**, 1431-1433

9. Shuttleworth, D. Preparation of metal-polymer dispersions by plasma techniques. An ESCA investigation. *J. Phys. Chem.* **84**, 1629-1634 (1980).
